# Identification and Analysis of Intermediate Size Noncoding RNAs in the Human Fetal Brain

**DOI:** 10.1371/journal.pone.0021652

**Published:** 2011-07-18

**Authors:** Dongsheng Yan, Dandan He, Shunmin He, Xiaoyan Chen, Zhen Fan, Runsheng Chen

**Affiliations:** 1 Bioinformatics Laboratory and National Laboratory of Biomacromolecules, Institute of Biophysics, Chinese Academy of Sciences, Beijing, China; 2 School of Ophthalmology and Optometry, Eye Hospital, Wenzhou Medical College, Wenzhou, Zhejiang, China; 3 Graduate School of the Chinese Academy of Sciences, Beijing, China; 4 Key Laboratory of the Zoological Systematics and Evolution, Institute of Zoology, Chinese Academy of Sciences, Beijing, China; Massachusetts General Hospital, United States of America

## Abstract

The involvement of noncoding RNAs (ncRNAs) in the development of the human brain remains largely unknown. Applying a cloning strategy for detection of intermediate size (50–500 nt) ncRNAs (is-ncRNAs) we have identified 82 novel transcripts in human fetal brain tissue. Most of the novel is-ncRNAs are not well conserved in vertebrates, and several transcripts were only found in primates. Northern blot and microarray analysis indicated considerable variation in expression across human fetal brain development stages and fetal tissues for both novel and known is-ncRNAs. Expression of several of the novel is-ncRNAs was conspicuously absent in one or two brain cancer cell lines, and transient overexpression of some transcripts in cancer cells significantly inhibited cell proliferation. Overall, our results suggest that is-ncRNAs play important roles in the development and tumorigenesis of human brain.

## Introduction

It is increasingly becoming evident that a major part of the information content in eukaryote genomes is expressed in the form of non-protein-coding RNAs (ncRNAs; [1]). The advancements in the ncRNA field, including the discovery of novel classes of ncRNA as well as new members of existing classes, steadily expand our understanding of ncRNA function. NcRNAs have been demonstrated to act as key regulators in most aspects of cellular and developmental biology, including embryonic development, cell differentiation and tumorigenesis [2], [3], [4].

A variety of different noncoding transcripts are involved in development and function of the vertebrate central nervous system [5]. MicroRNAs remain the most intensively investigated noncoding segment, and a number of brain specific miRNAs with distinct functions have been investigated [6]. Some microRNAs are highly expressed in vertebrate neural tissues [7], and Dicer mutants in mice and zebrafish show various neurological phenotypes [8], [9]. In worm and fly model organisms miRNAs decide neuron sensory asymmetry [10] and regulate sensory organ development by specifying the generation of neuronal precursor cells [11]. MicroRNAs have also been associated with a several neurodegenerative diseases such as Alzheimers' dementia [12], [13], Huntington's disease [14], and glioblastoma [15].

Despite the intense focus on miRNAs in recents years, these transcripts represent only a fraction of the entire non-protein coding transcriptional output from mammalian genomes. Far less attention has been directed at the possible involvement of other ncRNAs on brain development and function. Nonetheless, analysis of pig noncoding EST (expressed sequence tag) expression patterns indicated a higher frequency of candidate ncRNAs being expressed in central nervous system relative to other tissues; in contrast, the testis showed a high number of expressed protein coding genes, but a relative low number of non-coding transcripts [16]. In the mouse a high number of longer ncRNAs are expressed in the central nervous system [17]. The conserved ncRNA *TUG1* is expressed in the eye, brain and a few other tissues, and is required for photoreceptor development in the mouse [18]. The brain cortex expressed *HAR1F* and *HAR1R* transcripts originate from a locus that is highly conserved in all mammals, including the chimpanzee, but show distinct differences in primary sequence and secondary structure in human [19]. The maternally expressed ncRNA *Meg3/Gtl2* is expressed in the mouse brain, inner ear and eye, displaying differently spliced isoforms in the different tissues [20], and in response to infection by several encephalitic viruses the 3.2 kb noncoding VINC locus is expressed in the mouse brain [21].

There are fewer examples of involvement of intermediate size (i.e., 50–500 nt) ncRNAs in neuronal processes. A few small nucleolar RNAs are exclusively expressed in mouse and rat brain [22], [23]. Homologues to three of the mouse loci map to the human chromosomal region 15q11-q13, which contains a large number of tandemly repeated C/D box snoRNA loci with paternal imprinted expression [22]. This chromosomal region is implicated in the neurogenetic Prader-Willi syndrome (PWS) [22], and detailed analysis suggests the *PWCR1/HBII-85* snoRNA cluster and the single *HBII-438A* snoRNA as the most likely candidate loci involved in the syndrome [23]. The rat C/D box snoRNA RBII-36 locus is situated within an intron of the noncoding RNA *Bsr*, and appears to be generated from both debranched intron-lariats as well as from endonucleolytic cleavage of the *Bsr* primary transcript [24]. Another example of intermediate-size brain specific noncoding RNAs are the *BC1* and *BC200* transcripts found in rodents and primates, respectively. These transcripts have cytoplasmic neuronal expression patterns, including a concentration to dendrites [25], [26] and axons [27]. Both RNAs bind to several proteins involved mRNA translation [28], [29], [30], and their expression appear to depend on neuronal activity [31]. Knock-out *BC1* mice do nonetheless develop normally, but show signs of increased anxiety [32].

In the present study, we report a systematic identification and validation of 82 novel intermediate-size ncRNAs (is-ncRNAs) from human fetal brain. These novel is-ncRNAs are generally not well conserved in vertebrates, and several loci are only found in primates. Northern blot and microarray analysis indicated considerable variation in is-ncRNA expression across fetal tissues and fetal brain development stages, and expression of several novel is-ncRNAs were dramatically decreased in one or two brain tumor cell lines. Moreover, transient overexpression of some is-ncRNAs in SH-SY5Y cells significantly affected cell vitality and proliferation. Altogether, our results provide new insights into the diversity of is-ncRNAs and their involvement in brain development and tumorigenesis.

## Results

### ncRNA-specific library

**Figure 1:**
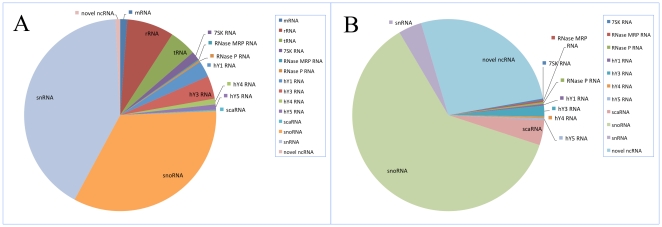
Distribution of sequenced clones and is-ncRNA loci. (A) Distribution of sequenced clones. The segment denoted as “novel ncRNAs” refers to previously unreported transcripts. (B) Distribution of genetic loci corresponding to the detected is-ncRNAs.

**Figure 2:**
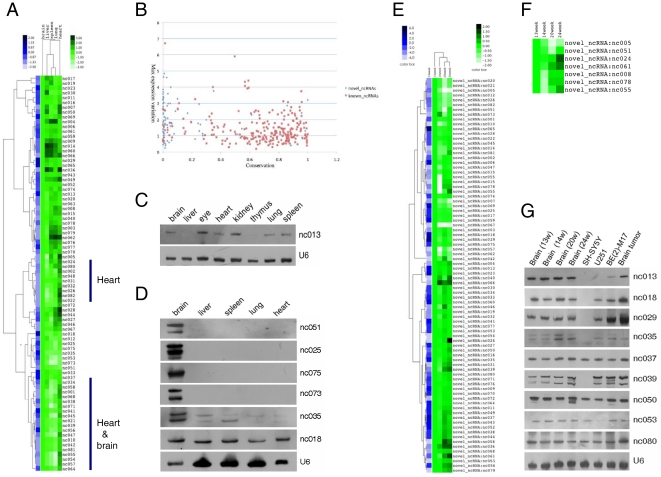
Expression of novel is-ncRNAs in human fetal tissues. (A) Relative expression of 82 novel is-ncRNAs extracted from human fetal brain tissue as analysed by microarray. The leftmost column (shades of blue) shows the expression levels of different novel ncRNAs in fetal brain tissue relative to the average expression in this tissue. The five columns to the right (shades of green) show the expression of each novel ncRNA relative to its expression in fetal brain. (B) Relationship between conservation and expressional variation among fetal tissues. The figure shows PhastCons scores plotted against maximal expressional variation among fetal tissues of known (red) and novel (blue) is-ncRNAs. (C) Northern blot of the novel is-ncRNA nc013. Note the lack of expression in liver and thymus. (D) Northern blot analysis of 6 novel transcripts with predominant or unique expression in the human fetal brain. (E) Relative expression of 82 novel is-ncRNAs in human fetal brain during four gestation stages. The leftmost column (shades of blue) shows the expression levels of different novel ncRNAs in fetal brain tissue at 13 weeks of gestation. The four columns to the right (shades of green) show the expression of each novel ncRNA in fetal brain at gestation stages 14 weeks, 20 weeks and 24 weeks relative to its expression at 13 weeks of gestation. (F) A magnified view from Figure E, which represents the relative expression of 7 novel is-ncRNAs in human fetal brain during four gestation stages. (G) Expression of nine novel is-ncRNAs expression during fetal brain development, in three tumor cell lines and in one clinical brain tumor sample. snRNA U6 served as an internal loading control.

To discover novel human ncRNAs , we used a previously described strategy [33] to construct a ncRNA-specific full length library from human fetal brain. Intermediate size ncRNAs (50–500 nt) were extracted from human fetal brain tissue, cloned and sequenced (Materials and Methods). A computational pipeline was developed to process the sequencing data and to distinguish novel ncRNA candidates from known classes of small RNAs. Removal of sequences that represented annotated mRNAs, rRNAs and tRNAs from the altogether 20,539 sequenced clones left 17,723 transcripts corresponding to 326 unique sequences which could be mapped to 331 loci in the human genome (Figure 1A; Figure S1; Table S1). The majority of these (244 unique sequences or 249 loci) corresponded to known or predicted (henceforth referred to as “known”) is-ncRNA genes of various classes, whereas 82 unique sequences/loci did not map to any annotated gene, and were consequently considered as potential novel ncRNA candidates (Figure 1B). The majority of these 82 unique sequences were identified by a single clone, and may represent RNA species that function at low copy number or in only a limited number of cell types. Northern blot or RT-PCR analyses gave positive signals for all of the 82 sequences in brain tissue (Figure 2; Figure S2 and S3).

### Genomic location of the novel ncRNAs

Mapping the genomic loci of the 82 novel ncRNAs revealed no significant deviation from a random chromomal distribution (Table S2). There were 27 intergenic loci, 44 and 11 loci that located in sense or antisense orientation, respectively, to introns of protein-coding genes, and the host genes of the 44 sense intronic loci were analysed for enrichment of annotated gene ontology (GO; [34]) or cellular pathway (KEGG; [34]) terms. No GO function term was statistically enriched among the host genes; however, four of the 44 host genes belong to the ‘Axon Guidance’ pathway (p-value = 4.04×10^−4^; details concerning host gene annotation and position in the pathway are found in Table S3). Of the corresponding four ncRNAs, nc068 showed a ‘brain and heart specific’ expression pattern (Figure 2A). Two of the ncRNAs (nc012 and nc026) also displayed developmentally related changes in fetal brain expression (Figure 2E). As no sequence homology extending 10 bp was found between any of these four ncRNAs and the coding regions of their respective host genes, any potential co-regulatory relationships between the ncRNAs and their host genes is probably not based on extensive Watson-Crick basepairing.

Several of the novel ncRNA loci overlap loci annotated as “noncoding”, and may represent active loci with sequence or secondary characteristics that deviate from what is commonly found in the human or other mammalian genomes. Potential snoRNA and scaRNA candidates were identified by applying the snoScan/snoGPS, snoSeeker, and snoReport software [35] to the longest sequence read from each locus. Fourteen transcripts with clear snoRNA or scaRNA characteristics were identified (Table S4), of which four were identified as C/D box snoRNAs, nine were H/ACA box snoRNAs, and one transcript (nc082) which showed both C/D box and H/ACA box characteristics is a likely scaRNA candidate.

### Conservation

**Figure 3:**
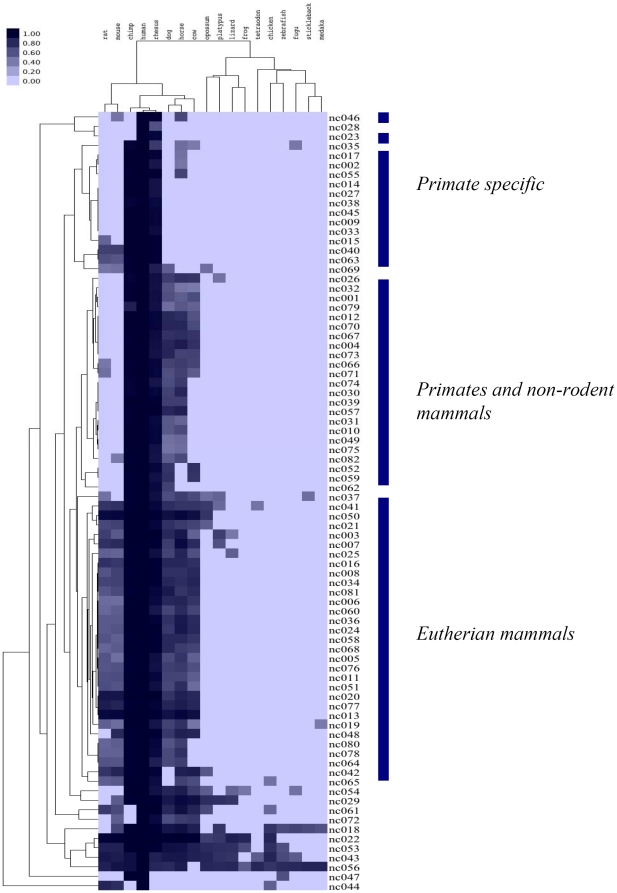
Conservation of 82 novel human is-ncRNA loci in 17 different vertebrates. The scale indicates average PhastCons score. As indicated, most novel is-ncRNA loci could be broadly divided into three groups based on similar conservation characteristics.

To assess the conservation level of the 82 novel is-ncRNAs we utilized the 28-way vertebrate sequence alignment by Miller [36], but restricted our analysis to the 18 species (including human) with a more than five-fold overall sequence coverage. As strongly conserved ncRNA loci would already have been detected by sequence comparisons, it was expected that most of the novel candidate loci would show limited sequence conservation. In accordance with this expectation, only 5 of the novel loci were conserved beyond eutherian mammals (Figure 3). This group nonetheless contained two of eight transcripts (nc018 and nc055) with predominant expression in the fetal brain, suggesting a link between is-ncRNAs and central nervous system development extending all the way back to the fish.

The majority of the novel is-ncRNA loci could be broadly clustered into three groups based on similar conservation characteristics (Figure 3). The largest group contains 31 loci that are conserved in most eutherian mammals, but not beyond these. Most of these show strong conservation (average PhastCons score >0.8) only in primates, and only two loci are strongly conserved throughout eutherian *Mammalia*. Both of these display strong tissue specific expressional characteristics; nc050 having distinctly elevated expression in liver and heart, and nc013 being conspicuously absent in two (liver and thymus) of eight analysed tissues (Figure 2). An additional point of note is that most of the novel ncRNA loci tend to be more strongly conserved in the cat, horse and dog than in the rodent genomes.

A second cluster is composed of 22 loci that are conserved in primates and most non-rodent mammals, but not in mouse or rat. This is a peculiar conservation pattern given that recent phylogenies [36] place rodents closer to the primate branch than for instance the carnivores (although not without debate [37]), and might suggest a more divergent development of is-ncRNAs in rodents relative to other mammals. This is supported by the overall tendency towards a higher conservation levels in the cow, horse and dog genomes than in the rodents. As in the preceding group, strong conservation is mostly limited to the primates, and the average PhastCons score of these transcripts in other mammals is generally below 0.6. Together with the transcripts only conserved in primates, this group is enriched in loci with distinctly differential expression in fetal tissues (Figure 2).

The third conservation group is a cluster of 14 loci that are well conserved in the three primates included in the analysis (human, chimpanzee and rhesus macaque), and a few loci showing either conservation only between human and one of the primates (nc023, nc028, nc046 and nc047) together with one transcript conserved only in rodents (and possibly also chicken; nc044). A subgroup of the primate-conserved transcripts include four loci (nc015, nc040, nc063 and nc069) that are also conserved in at least one of the rodents, but generally this cluster shows little conservation beyond the primates.

To obtain a more complete view of the conservation state of the novel is-ncRNAs in the primate lineage, we carried out a BlastN on all the five primates for which genomic sequence data are presently available (human, chimpanzee, orang-utan, rhesus macaque and marmoset monkey; Figure S4). The BlastN data show that the bulk of the novel transcripts is generally conserved across the primate lineage. However, of the core ‘primate specific’ novel is-ncRNAs (i.e. those not even conserved in the rodents), about half (5/11) of the loci are not conserved in the New World marmoset monkey, suggesting that these loci have either appeared in the Old World lineage after the split from the New World monkeys or are too diverged to be identified by sequence comparison. Pending the quality and completeness of the ape genomes, the data also confirm that the nc028, nc044 and nc046 loci have arisen (or evolved quickly) in the human lineage, since these three loci are not conserved in any of the two greater apes, and each show possible conservation in only one of the two monkey genomes.

### is-ncRNA expression profile

The expressional characteristics of the 82 novel and the 244 known is-ncRNAs detected in the study were examined by Northern blot and microarray analysis in several fetal tissues (brain, heart, liver, lung and spleen), and during four stages of human fetal brain development.

Expressional distribution of is-ncRNAs was studied in the fetal tissue at the 24-week gestation stage. While the majority of both known and novel transcripts showed only minor differences in expression levels, more than one third (34) of the novel transcripts showed distinct (i.e. >2-fold) differences in expression among different tissues (figure 2A and Figure S5). These included transcripts with distinctly elevated expression in a single tissue (e.g. liver, nc030) or in two or more tissues (e.g. spleen and lung, nc004), and transcript whose expression is strongly reduced in one (e.g. nc071, spleen) or a few tissues (nc045). The expression of transcript nc013 was investigated further by Northern blot analysis, and was found absent in liver and thymus despite being clearly expressed in an extended number of tissues (Figure 2C). Relating expressional characteristics to conservation status suggested a tendency towards novel ncRNA loci with strong expressional variation across fetal tissues being less conserved (i.e. conserved only in primates and/or a few other mammals) than loci with more even expression patterns (Figure 2B).

Hierarchical clustering of the novel ncRNAs divided the transcripts into two approximately equal groups with respect to their expressional patterns. The first group (upper half of Figure 2A) were composed of transcripts with some level of expression in most tissues, whereas the second group suggested the existence of several sub-groups of transcripts with expression mainly limited to one or two tissues. These included one sub-group comprised of 8 transcripts with predominant expression in the heart, and an additional sub-group of 22 transcript with low expression levels in most tissues other than heart or brain, along with a third sub-group consisting of transcripts with predominant expression in the brain.

Given the importance of the brain in human evolution, we were particularly interested in ncRNAs with brain specific expression. The microarray data indicated that the expression of a number of both known and novel transcripts was higher in the fetal brain than in other tissues (Figure S5). These included several snoRNAs belonging to the imprinted HBII-52 cluster, which is known to be highly expressed in mammalian brain and recently implicated in the Prader-Willy syndrome pathology [23]. However, several other annotated is-ncRNAs (e.g. snoRNAs U105B, U34 and U80) which have previously not been reported as brain specific also showed predominant or exclusive expression in the fetal brain at the tested gestation stage. Of the novel ncRNAs with specific or predominant expression in the fetal brain, we obtained Northern blotting results across five tissues for 6 of the transcripts. Four of these were only expressed in the fetal brain, whereas the two remaining transcripts showed elevated expression in this tissue (Figure 2D). The data thus strongly suggest that specific expression of intermediate-size ncRNAs is an aspect of fetal human brain development.

When all analysed is-ncRNAs (i.e. known and novel) are compared, the expression levels of known loci generally appeared to be higher than those of the novel loci, whereas the expressional variation across tissues was more pronounced for the novel loci (Figure S5). To the extent that known loci did show distinct expressional variation across tissues, the majority of these showed elevated expression in the brain relative to other tissues (e.g. the HBII-52 series of snoRNAs). In total only 46 (19%) of the known transcripts show more than 2-fold expressional variation between tissues, compared to 41% of the novel transcripts. It thus appears that previously known is-ncRNAs generally represent highly and ubiquituously expressed noncoding loci, whereas the set of novel transcripts obtained in our approach to a larger extent may represent less abundant transcripts with a higher tendency towards variable expression across different tissues. Hierarchical clustering of all investigated transcripts produced a clustering pattern with only partial resemblance to that observed for the novel transcripts. Intriguingly, the clusters of novel transcripts with predominant expression in the heart, or in heart and brain, remained mostly intact when analysed along with previously known transcripts, suggesting that these may represent previously uninvestigated expression patterns of human is-ncRNAs. On the other hand, the novel transcripts with predominant expression in the fetal brain disperse among several clusters dominated by known is-ncRNAs, possibly reflecting earlier efforts on mapping such transcripts in the human or mammalian central nervous system [38].

Northern blot analysis of a small number of the novel is-ncRNAs across four gestation stages (13, 14, 20 and 24 weeks) showed clear differences in fetal brain expression levels for at least two of the transcripts (Figure 2G). Further analysis by microarray showed various expression patterns across these four gestation stages (Figure 2E). Only seven of the novel transcripts (8.5%) displayed distinct (i.e. >2-fold) variation in their expression levels through the course of the investigated period. However, when contrasted with the expressional variation produced by the 245 known is-ncRNAs, of which only one transcript (snoRNA U14-5; Figure S3) showed distinct expressional variation across the same developmental period, this nonetheless suggests that the novel set of is-ncRNA may be enriched in transcripts related to fetal brain development. Given that the expressional analysis only includes four time points over less than one-third of the gestation period, and that many (∼14) show very low expression throughout the period, the novel set of is-ncRNAs might well contain an even higher number of transcripts that are activated during other times of fetal brain development.

The seven novel transcripts with distinctly differential expression levels across gestation stages display varying expression patterns. Transcripts nc024 and nc061 show inclining expression over the analysed period, peaking at week 24, whereas transcripts nc005 and nc051 decline towards week 20, thereafter rising sharply. The remaining three novel transcripts all show an expression minimum at week 14, followed by increased expression at the two later stages. The previously annotated snoRNA U14-5 is apparently activated at some stage after week 14, and show strong expression in week 20 to 24. The seven novel transcripts are with one exception (nc055) conserved in most placental mammals (Table 1), which should allow for functional studies of homologues of these transcripts in rodents or other mammals. The primate specific nc055 is found in the genomes of all sequenced primates apart from the marmoset, and a possible homologue also exists in the horse genome. Three of the transcripts (nc005, nc024 and nc055) show distinctly differential expression across tissues, but only transcript nc051 shows brain specific expression (as verified by Northern blot analysis), thus, their temporal activities may not necessarily be restricted to fetal brain development.

**Table 1:** Seven novel is-ncRNAs showing distinct (>2-fold) variation in expression across four gestation stages.

ID	Conservation	Tissue (>2-fold)
nc005	Placental mammals	Heart (up)
nc008	Placental mammals	Lung
nc024	Placental mammals	Heart, spleen (up)
nc051	Placental mammals	Brain
nc055	Primates	Heart, brain (up)
nc061	Placental mammals	All the tissues
nc078	Placental mammals	All the tissues

In comparison to the novel transcripts, the known is-ncRNAs were characterized by a generally higher overall expression level in the fetal brain, while simultaneously a large number of the transcripts showed very little (<1-fold) expressional variation throughout the investigated period (Figure S6). The snoRNA HBII-52 cluster loci, though not distinctly elevated at any stage, showed a general tendency to higher expression towards the end of the period, whereas the apparently brain-specific snoRNAs U105B, U34 and U80 all show intermediate and very even expression levels across the four gestation stages.

### Ectopic expression of is-ncRNAs inhibited cancer cell proliferation

Noncoding RNAs have been linked to tumor development in several cases [39], [40], [41], [42], and we therefore used Northern blotting to examine the expression of nine novel is-ncRNAs in three tumor cell lines and one clinical brain tumor sample. Five of nine tested novel is-ncRNAs were not expressed in the neuroblastoma cell line SH-SY5Y, and one was not expressed in the glioma cell line U251 (Figure 2G). In contrast, the BE(2)-M17 neuroblastoma cells and the clinical tumor sample displayed elevated expression of transcripts nc050 and nc029. Microarray analysis of cell lines SH-SY5Y and U251 further indicated that several known and novel transcripts showed strongly increased or reduced expression (Figure S7).

**Figure 4:**
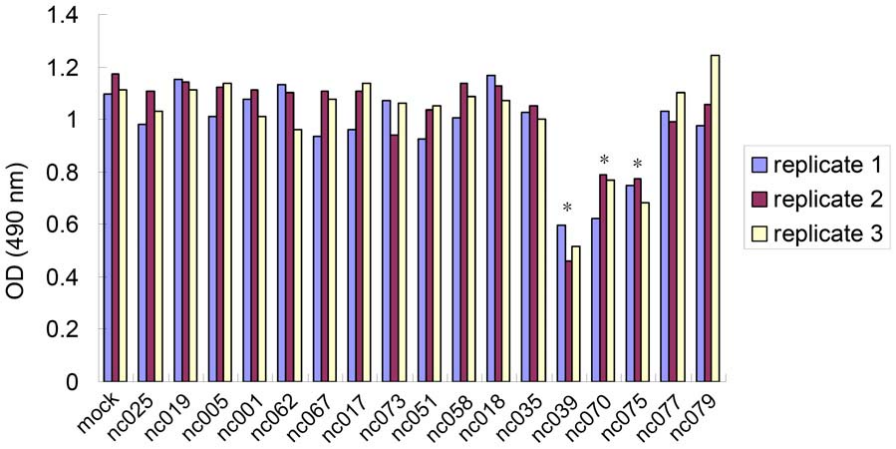
MTS assay of SH-SY5Y cells transfected with 17 different is-ncRNAs. MTS cell proliferation assay was carried out on day 3 after lipofectamine transfection of SH-SY5Y cells with is-ncRNAs constructs or a mock vector as negative control. As indicated, cell populations transfected with nc039, nc070 or nc075 construct had a significantly fewer number of metabolically active cells than cells transfected with a mock vector. The data are expressed as three replicates in one experiment. Results represent those obtained in three experiments.

Given the conspicuous absence of expression of several novel is-ncRNAs in the cell lines, we speculated whether this lack of expression might be directly related to cell vitality and proliferation. We therefore cloned 17 different novel is-ncRNA in the lentiviral expression vector pSIH1-H1-Puro, and studied the activity of SH-SY5Y cells after transfection with these vectors. Seven of the novel is-ncRNAs that were selected for this study were either predominantly expressed in brain (nc025, nc051, nc073, nc075) or not expressed in SH-SY5Y (nc039), or had both these expressional characteristics (nc018, nc035), whereas ten transcripts were randomly selected among the novel clones (nc001, nc005, nc017, nc019, nc058, nc062, nc067, nc070, nc077, nc079). The SH-SY5Y cells were cultured for 72 hours after transfection, and the effects on cell vitality were estimated using the MTS assay. Cells transfected with three of the 17 is-ncRNAs showed significantly reduced formazan concentration (53.63±3.47% decrease for nc039, 35.59±4.64% decrease for nc070 and 34.99±2.42% decrease for nc075, *p*<0.01, n = 3) compared to cells transfected with empty vector (Figure 4), and visual inspection of these cell cultures indicated markedly reduced cell densities. The reduction in cell activity was most pronounced in cells transfected with nc039, which was one of the transcripts whose expression was undetectable in the SH-SY5Y cell line, whereas transcript nc070 and the brain specific nc075 appeared to reduce cell activity to a lesser extent. Though preliminary, these data do suggest that among the numerous deviating expressional patterns observed in the carcinogenic cell lines, there may exist is-ncRNA activity that pertains directly to the carcinogenic state of these cells.

## Discussion

Recent analysis have demonstrated that the central nervous system is among the tissues most enriched in expressed miRNAs [43] and long noncoding transcripts [1] in mammals. Though the specific functions of most noncoding transcripts remain unknown, it has been suggested that bridging the informational gap between the analogue information of protein conformational space and the digital information of the genome may be a particularly important aspect of ncRNA function in the evolution and function of the central nervous system [44]. In the present study, we have obtained experimental evidence for 82 novel intermediate size ncRNAs expressed in the fetal brain, and although these transcripts do not constitute a large fraction of known human transcripts in this size range, they nonetheless provide additional insights into the noncoding transcriptome in the human fetal brain.

The study detected altogether 326 species of is-ncRNA, of which 75% were previously known (or predicted) transcripts. The expression levels of known and novel is-ncRNAs, as estimated from their clone numbers, were markedly different. Whereas a majority of the previously known is-ncRNAs was detected as multiple clones, most of the novel is-ncRNAs were detected as single or few copy clones. Known transcripts are commonly ‘housekeeping’ types of is-ncRNAs with general functions (e.g. splicing, RNA modification, etc) requiring relatively high expression levels in all or most cells. The novel is-ncRNAs, on the other hand, are probably most transcripts that are expressed at either lower overall levels or only in few cells or cell types, both suggesting more specific functional repertoires.

As also apparent in this study, is-ncRNAs loci are commonly found within the introns of protein coding genes, from which they may either be released during pre-mRNA splicing, or be transcribed from independent promoters [33]. Though intronic is-ncRNA loci may not be functionally related to their host genes, functional correlations are observed (e.g. snoRNAs are commonly found within introns of genes coding for ribosomal proteins [33]). The observation that host genes for the novel is-ncRNAs extracted from human fetal brain displayed statistically significant enriched in a cellular pathway related to neuronal development is thus intriguing, in particular as several of the hosted loci were preferentially expressed in fetal brain tissue or showed distinct expressional variation during fetal brain development.

Close to one-fifth of the novel transcripts are not extensively conserved beyond the primates, adding to the increasing number of primate-specific ncRNAs detected in recent years [45]. Although the functional aspects of such transcripts are only slowly being unravelled, there are already several indications of ncRNAs being involved in central nervous system development activity. The *HAR1F* RNA, transcribed from a highly conserved mammalian locus showing rapid evolvement in the human lineage, has been implicated in cortical development in human and chimpanzee [46].

The expressional characteristics of the detected is-ncRNAs were further examined by Northern blot and microarray analysis. The data showed distinct variations in expression levels for a subset of the novel transcripts, including eight transcripts with predominant expression in the fetal brain, and a larger group mostly expressed in brain and heart. The quality of the microarray analysis was corroborated by the fact that most is-ncRNAs known to be expressed in the brain showed distinctly elevated expression in the fetal brain relative to other tissues. The failure to clone the *BC200* transcript probably owes to its internal A-rich tract, which lead to its removal from the RNA extract along with the polyA-tailed RNA fraction. The observation that a number of novel transcripts cloned from fetal brain extracts nonetheless show predominant expression in tissue other than the fetal brain, could be explained by is-ncRNAs having positive functions in various tissues across a wider range of expressional levels. It could also be that a given is-ncRNA may have functions within a limited range of specific cell types distributed among several tissues, thus appearing to occur at relatively low levels in tissues where it is only expressed in a minority of the cells or cell types constituting the tissue.

Though the expressional tissue specificity of the novel is-ncRNAs did not strongly reflect their fetal brain origin from which they were cloned, transcripts showing distinct changes in expression levels during fetal brain development were relatively more frequent among the novel is-ncRNAs (7 out of 82) than in the collection of previously known transcripts (1 out of 244). The functional relevance of the developmental changes in is-ncRNA expression remains to be elucidated for both known and novel transcripts, but it nonetheless demonstrates the potential of focusing on this complement of the transcriptome for obtaining further knowledge on neuronal development and function. An especially intriguing case in this respect is represented by transcript nc051 which in addition to its evolutionary constraints and particular genomic location also display distinct variation during fetal brain development. The single known transcript showing distinct expressional variation through fetal development (snoRNA U14-5) adds to the increasing number of snoRNA-like transcripts apparently engaged in roles not readily explained by the mere ‘housekeeping’ modifications of rRNA, tRNA or snRNAs. The involvement of snoRNA-like transcripts in CNS (central nervous system) function is increasingly attracting attention. The complement of snoRNA-like transcripts in mammals and other vertebrates [47] is much larger than can be accounted for by the traditional roles of such transcripts in modification of rRNA, tRNAs and snRNAs. The functional roles of these ‘orphan’ snoRNA-like transcripts are largely unknown, though snoRNA involvement in the regulation of pre-mRNA splicing has been demonstrated in one case concerning the imprinted HBII-52 locus [48]. Six of the novel is-ncRNAs show distinct snoRNA-like characteristics, and three of these were preferentially expressed in brain, or brain and heart tissue.

The developmental expression analysis also provides additional information on the expressional characteristics of previously known and functionally described is-ncRNAs. The lack of variation in expression of such transcripts during fetal development is conspicuous, but might imply that the majority of these perform constitutive roles in neuronal tissue that operate from early stages of development. This might explain the relatively high expression levels of some of these is-ncRNAs (e.g. the HBII-52 snoRNAs) and their early identification as brain-expressed transcripts [24], [38]. Alternatively, such transcripts might reach their peak activity at developmental stages outside the temporal range investigated in this study.

In addition to functions in brain evolution and development, ncRNAs have been implicated in CNS disorders or malignancies. MicroRNA-21 is highly expressed in glioma and targets various genes involved in cell proliferation, migration and apoptosis [15], [49], and has been linked to glioma development [15], [49]. On the other hand, miR-124a, the most abundant miRNA in central nervous system, has been reported to be down-regulated in high-grade glial neoplasms and suppress glioma cell proliferation [50]. The Alu-derived *BC200* ncRNA has long been studied for its potential role in regulation of post-synaptic translation, possibly in concert with the fragile X mental retardation protein [51], and knock-down of its putative rodent analogue *BC1* produces behavioural deviations in mice [32]. *BC200* also displays expressional increases that correlates with spatial and progressive severity of Alzheimer's symptoms [52], and is overexpressed in a number of non-neuronal malignancies [53]. The snoRNA-encoding GAS5 has also been implicated in cancer development [54]. Several of the novel is-ncRNAs were shown to be absent or down-regulated in SH-SY5Y cells, as compared with normal brain tissue, and restoration of three transcripts expression resulted in a pronounced decrease in the cell numbers. Although this evidence is still preliminary and incomplete, it does nonetheless imply that these is-ncRNAs are involved in the cell proliferation process, and possibly also associated with tumor development. Future research on cause and maintenance of malignant cell states should focus further on this specific complement of noncoding transcripts.

In summary, the novel is-ncRNAs reported in this study represent an important early step in appreciating the significance of ncRNAs in human brain biology. Moreover, our results not only identify a number of ncRNAs that may be subjected to future study, but also support the notion that some of these novel transcripts are intrinsically functional and involved in brain development and tumorigenesis.

## Materials and Methods

### Cell lines

Three human tumor cell lines, SH-SY5Y, BE(2)-M17 and U251, were purchased from the Institute of Basic Medical Sciences of the Chinese Academy of Medical Sciences. SH-SY5Y cells were cultured in DMEM/F-12 1∶1 (Invitrogen), and the two other cell lines were cultured in Dulbecco modified Eagle medium (DMEM) supplemented with 10% fetal bovine serum (Hyclone, Logan, UT) and L-glutamine at 37°C. All cells were incubated at 37°C in a humidified incubator containing 5% CO_2_.

### Ethics statement

We collected human fetal brain tissue from 5 gestational stages (12 weeks, 13 weeks, 14 weeks, 20 weeks and 24 weeks) and human fetal liver, spleen, heart and lung tissues at 24 weeks. Sample collection was approved by the Wenzhou Medical College ethics committee on research involving human subjects, and written informed consent was obtained from the parents in each case. All experiments were performed in compliance with the Helsinki Declaration and national laws.

### ncRNA library construction

The ncRNA libraries were constructed as previously described [33]. Total RNA was isolated from the human fetal brain tissues according to the Trizol (Invitrogen) protocol. 1 mg total RNA from 4 different gestational stages (12 w, 14 w, 20 w, 24 w) was pooled and mixed in equal aliquots, then the total RNA mix was loaded on a Qiagen RNA/DNA maxi column (Qiagen). The column was maintained at 50°C in a water bath and the RNA was eluted on a 0.7–1.1 M NaCl gradient in QRV2 buffer (Qiagen RNA/DNA Handbook). Each of the eluted fractions were isopropanol precipitated with glycogen and 3 M NaAc (pH 5.2) overnight at −20°C. The 0.9 and 1.0 M NaCl fractions containing RNAs in the size range 50–500 nucleotides were pooled. Poly(A)^+^ RNA was removed using the Ambion poly(A) purist MAG Kit (Ambion). rRNAs, U1 and U4 snRNAs were removed using the MICROBExpress Kit (Ambion), as described in the kit manuals. The purified RNA was dephosphorylated with calf intestine alkaline phosphatase (CIAP, Fermentas) and ligated to the 3′ end adaptor oligonucleotide (see Supplementary Materials S1) using T4 RNA ligase (Fermentas). The ligation product was purified with Trizol and divided into two equal aliquots. One aliquot was treated with T4 Polynucleotide kinase (PNK, Fermentas) to phosphorylate the uncapped RNA species in the sample, the other aliquot was treated with Tobacco acid pyrophosphatase (TAP, Epicentre) to remove 5′-end methyl-guanosine caps from capped RNA species. After removal of the enzyme, each aliquot was ligated to the 5′ end adaptor oligonucleotide. After each ligation step, excessive adaptors and short fragments were cleaned up on a Qiagen RNAeasy mini elute column (Qiagen). The final pool of small RNA was reverse-transcribed (RT) with SuperScript III (SS III, Invitrogen) and 3RT primer (complementary to the 3′ adaptor oligonucleotide) at 50°C. The cDNA was amplified by polymerase chain reaction (PCR) using Platinum Taq (Invitrogen) with the 3RT and 5CD primers (see Supplementary Materials S1) for 15 or 25 cycles (94°C 15 sec, 50°C 30 sec, 72°C 30 sec). The PCR products were purified with the QIAquick PCR Purification Kit (Qiagen) and ligated into the TOPO PCR 2.1 vector (Invitrogen). Transformation into *E. coli* was performed with the Invitrogen ElectroMAX DH10BT1 electro transformation kit. About 25,000 clones were selected for Sanger sequencing at the Beijing Genomics Institute.

### Northern blotting

DIG-labeled RNA probes were *in vitro* transcribed from plasmid DNA with the DIG RNA Labeling Kit SP6/T7 (Roche). Total RNA were extracted from human fetal brain at 4 different gestational stages (13 w, 14 w, 20 w, 24 w), from human fetal liver, lung, spleen heart, thymus, kidney and eye at 24 weeks of gestation, and from 3 tumor cell lines (SH-SY5Y, BE(2)-M17, and U251). Total RNA was size-separated by 6% denaturing PAGE gel electrophoresis, transferred onto a nylon membrane (Hybond-N+; GE Healthcare), hybridized with the DIG-labeled RNA probes in RNA EASY Hyb buffer (Roche) at 60°C to 68°C overnight, and then treated with Blocking and Washing Buffer (Roche). After equilibration in detection buffer, blots were incubated with the chemiluminescent substrate CDP-star and exposed to Kodak Biomax MR film. A DIG-labeled U6 RNA probe was used as an internal control.

### RT-PCR

To confirm the expression of some ncRNAs in human fetal brain, RT-PCR was performed by the SuperScript® III First-Strand Synthesis System (Invitrogen), according to manufacturer's instructions. Specific primers were designed based on the sequences for each ncRNA. The PCR reaction was first at 94°C for 5 min. Thirty-five PCR cycles were performed with each cycle at 94°C for 15 seconds, 55°C for 30 seconds, and 72°C for 60 seconds. The final cycle was run in additional 10 minutes at 72°C. The PCR products were electrophorezed on PAGE gels, then stained with SYBR Gold nucleic acid gel stain (Invitrogen) and were photographed under blue-light transilluminators. The size of each PCR product was estimated using a standard 25 bp DNA ladder. Negative controls were also run simultaneously to achieve the accuracy of RT-PCR. Non-DNASE-treated samples containing genomic DNA were used as a template. The RT step was omitted in order to check DNASE-treated samples without residual genomic DNA. Each of the RT-PCR products was identified by DNA sequencing.

### 5′ -and 3′-RACE

Total fetal brain RNA was ligated to 3′ and 5′ adapters, and then reverse transcribed (RT) using SuperScript III. 5′-and 3′-RACE was performed by PCR amplification of the RT products, with one primer designed specific to the ncRNA sequence and the other primer corresponding to either the 5CD or 3RT adapter for the 5′- and 3′-RACE, respectively.

### Microarray sample preparation and hybridization

Human fetal brain tissues (13 w, 14 w, 20 w, 24 w) and fetal liver, spleen, heart and lung tissues (24 weeks) were used in the microarray analysis. Total RNA was dephosphorylated with calf intestine alkaline phosphatase (Fermentas) and ligated to the same 3′ adapter oligonucleotide that was used for library construction. 1 µg ligated RNA of each sample was used to create cRNA according to the protocol of the Low RNA Input Linear Amplification Kit (Agilent Technologies) with the minor modification that the ligated ncRNAs were reverse transcribed using an oligonucleotide complementary to the 3′ adapter (T7P-3AD2) and extended with a 5′ end T7 promoter sequence extension. The cRNA was purified with the mirVana miRNA isolation kit (Ambion), and labeled with Cy3 (or Cy5) using the CyDye mono-reactive NHS Esters kit (GE Healthcare). The labeled cRNA was purified by Qiagen RNeasy Mini Kit (Qiagen).

The microarray slides were prehybridized at 42°C for 1 h and hybridized at 42°C for 16 h. Microarrays were scanned using Axon GenePix 4000B (Molecular Devices), and raw data were quantified with the ImaGene 3.0 software.

### Microarray design and analysis

Microarray probes of 50 nt average length were designed against the 326 cloned and sequenced fetal brain ncRNAs. For ncRNAs longer than 200 nt two different probes were designed. In addition to probes against the 326 ncRNAs the microarray also contained positive control probes that were used to estimate the RNA hybridization efficiency. Each probe was printed in triplicate on a microarray (CapitalBio Corporation). The microarray was used to examine ncRNA expression in different fetal tissues and in fetal brain at various gestation stages. The analyses were based on the loop design method [55], [56], in which all combinations of two samples (from either different tissues of different gestations stages) were simultaneously hybridized to a microarray, each of the two samples labeled with a different fluorescent probe (Cy3 or Cy5). The microarray data were normalized using standard global media normalization [57]. Differential expression between tissues and gestation stages were calculated as log_2_-ratio using a method by Vinciotti *et al.* [57]. The data was hierarchically clustered based on the average-linkage methods, using the Cluster software [58], and visualized using TreeView [59].

### MTS assay

The ncRNAs nc067, nc035, nc025, nc017, nc039, nc019, nc073, nc070, nc005, nc051, nc075, nc001, nc058, nc077, nc062, nc018 and nc079 were cloned between the BamHI and EcoRI sites of the pSH1 vector (containing a puromycin resistance gene). The recombinant constructs and a mock vector (self-ligated pSH1 vector without insert) were transfected into SH-SY5Y cells by lipofectamine 2000 according to the manual. After 72 hours culture, cell proliferation was measured using the CellTiter 96 AQ_ueous_ assay kit (Promega) according to the manufacturer's instructions. Briefly, the CellTiter 96 AQ_ueous_ One Solution Reagent was added to each well before incubation at 37°C for 3 hours. Cell proliferation was assessed by absorbance at 490 nm using a microtiter plate reader (Molecular Devices). The data were analyzed using the Student's *t* test. Statistical significance was accepted at *p *< 0.05.

### NCBI GenBank and GEO submission

The ncRNA sequence data in this study have been submitted to GenBank under accession nos. HQ292100-HQ292181. All microarray data is MIAME compliant and the raw data have been deposited in the Gene Expression Omnibus (GEO, http://www.ncbi.nlm.nih.gov/geo/) with accession number GSE26196.

## Supporting Information

Figure S1 **Flow chart for the process of ncRNA identification in human fetal brain.** Pipline of is-ncRNAs identification and confirmation in human fetal brain as indicated in the figure.
(TIF)

Figure S2 **Northern blot analysis of 58 ncRNAs in human fetal brain.** As indicated, 58 is-ncRNAs identified in human fetal brain were confirmed by Northern blot analysis. Most of all have a single band within the expected size range. In some case with multiple bands, at least one band within the expected size range.
(TIF)

Figure S3 **RT-PCR analysis of 31 ncRNAs in human fetal brain.** As indicated, RT-PCR products of 31 ncRNAs in PAGE gels. RT+ indicated reaction with reverse transcriptase and RT- indicated omission of reverse transcriptase from the reaction to exclude the possible contamination by genomic DNAs. All the RT-PCR products are within the expected size range.
(TIF)

Figure S4 **BlastN sequence alignments of novel is-ncRNAs in primate genomes.** (A) Alignments of all novel is-ncRNAs in primate genomes. (B) Alignments of ‘Primate specific’ novel is-ncRNAs in primate genomes.
(TIF)

Figure S5 **Clustered expression profiles of is-ncRNAs in different tissues.** Expression patterns of 326 clustered ncRNAs (the figure includes both novel and known ncRNAs) in human fetal brain, liver, spleen, lung and heart tissues.
(TIF)

Figure S6 **Clustered expression profiles of is-ncRNAs during human fetal brain development.** Expression patterns of 326 clustered ncRNAs (the figure includes both novel and known ncRNAs) during human fetal brain development.
(TIF)

Figure S7 **Clustered expression profiles of is-ncRNAs in tumor cell lines.** Expression patterns of 326 clustered ncRNAs (the figure includes both novel and known ncRNAs) in glioma cell line U251 and neuroblastoma cell line SH-SY5Y, as compared with normal brain tissue.
(TIF)

Table S1 **Distribution of sequenced clones.** Distribution of sequenced library clones on different RNA species and categories. Sequenced clone numbers and percentage of novel and known ncRNAs are indicated in the table.
(DOC)

Table S2 **Genomic location of the novel ncRNA genes.** The length and chromosome location of all the novel ncRNAs are presented in the table.
(DOC)

Table S3 **Novel ncRNAs involved in axon guidance pathway.** Four ncRNAs, their host genes and annotations are shown in the table.
(DOC)

Table S4 **Predicted snoRNAs or scaRNAs of novel ncRNAs.** Fourteen ncRNAs with clear snoRNA or scaRNA characteristics were identified. As indicated, four ncRNAs were identified as C/D box snoRNAs, nine as H/ACA box snoRNAs, and one transcript (nc089) which showed both C/D box and H/ACA box characteristics is a likely scaRNA candidate.
(DOC)

Supplementary Materials S1 **Oligos used and all novel ncRNA sequences.**
(DOC)
