# Supplementary figures and images for: Identification and Analysis of Intermediate Size Noncoding RNAs in the Human Fetal Brain
Source: PLoS One. 2011 Jul 18;6(7):e21652. doi: 10.1371/journal.pone.0021652 (PMC3138756; doi:10.1371/journal.pone.0021652)

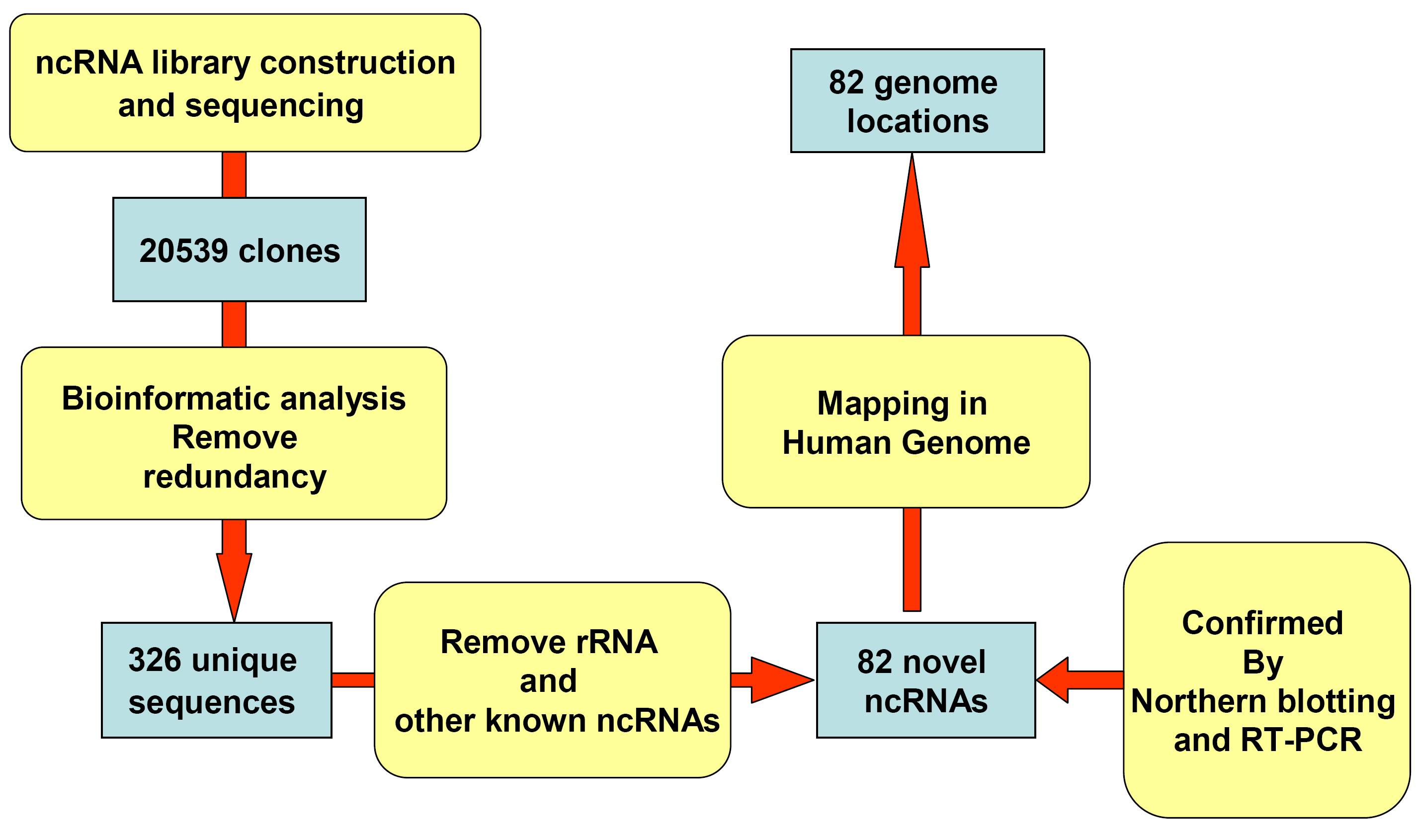

Supplement: Figure S1 — Flow chart for the process of ncRNA identification in human fetal brain. Pipline of is-ncRNAs identification and confirmation in human fetal brain as indicated in the figure. (TIF) [file pone.0021652.s001.tif]

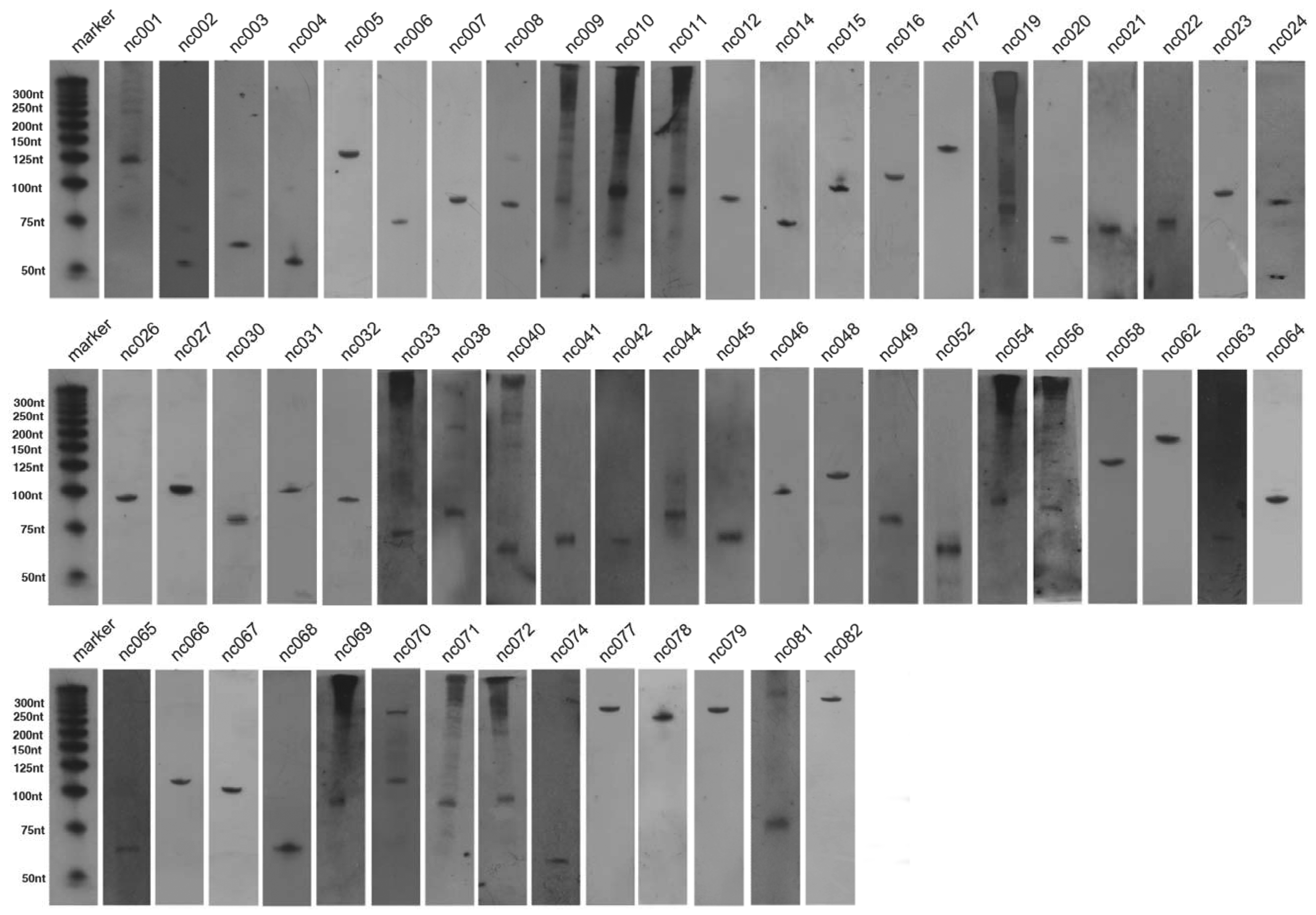

Supplement: Figure S2 — Northern blot analysis of 58 ncRNAs in human fetal brain. As indicated, 58 is-ncRNAs identified in human fetal brain were confirmed by Northern blot analysis. Most of all have a single band within the expected size range. In some case with multiple bands, at least one band within the expected size range. (TIF) [file pone.0021652.s002.tif]

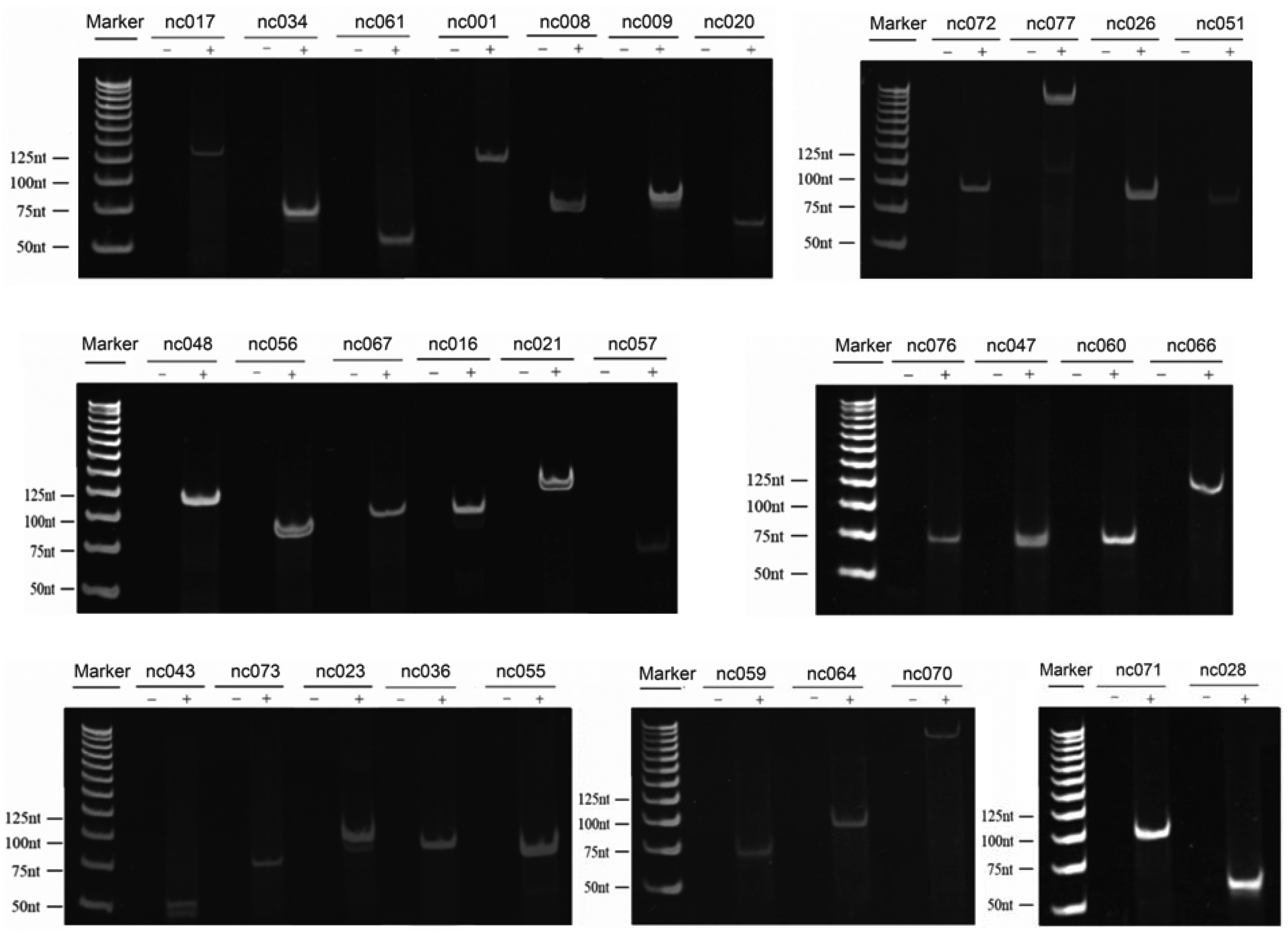

Supplement: Figure S3 — RT-PCR analysis of 31 ncRNAs in human fetal brain. As indicated, RT-PCR products of 31 ncRNAs in PAGE gels. RT+ indicated reaction with reverse transcriptase and RT- indicated omission of reverse transcriptase from the reaction to exclude the possible contamination by genomic DNAs. All the RT-PCR products are within the expected size range. (TIF) [file pone.0021652.s003.tif]

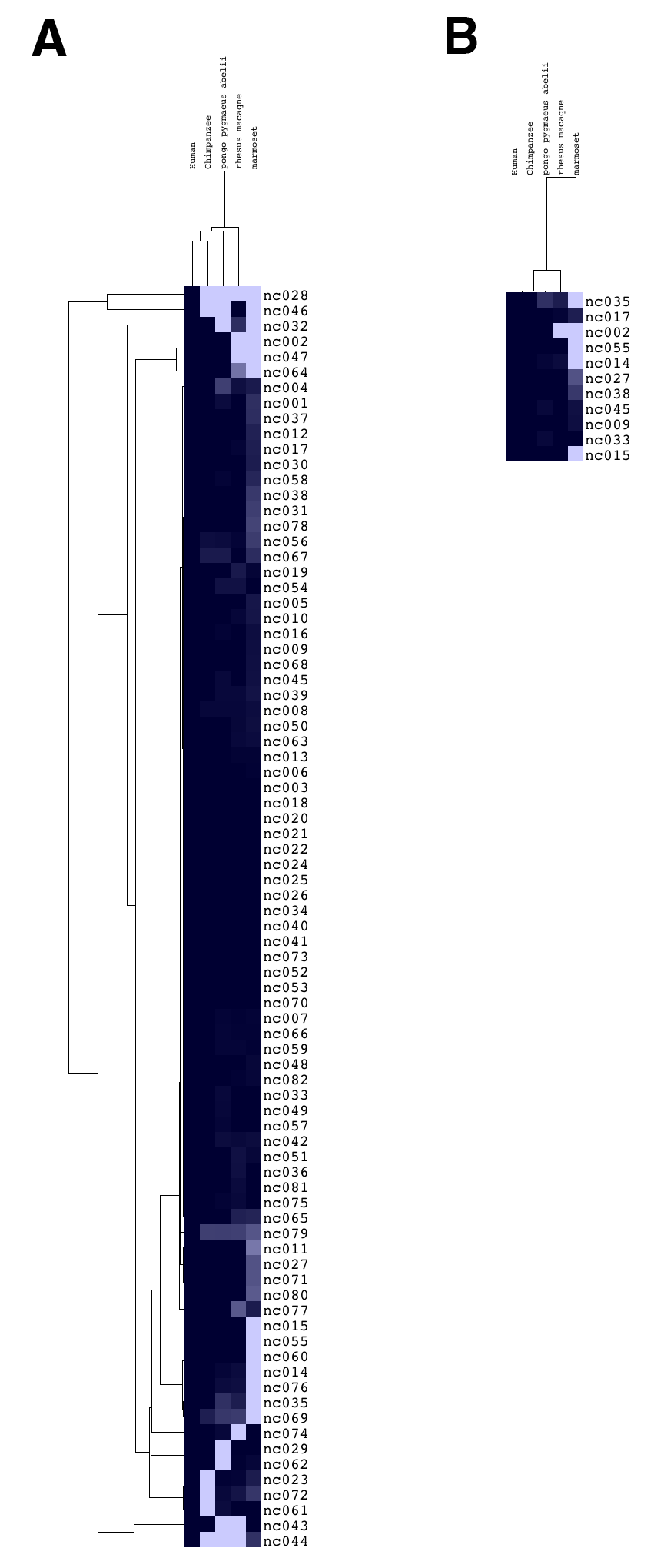

Supplement: Figure S4 — BlastN sequence alignments of novel is-ncRNAs in primate genomes. (A) Alignments of all novel is-ncRNAs in primate genomes. (B) Alignments of ‘Primate specific’ novel is-ncRNAs in primate genomes. (TIF) [file pone.0021652.s004.tif]

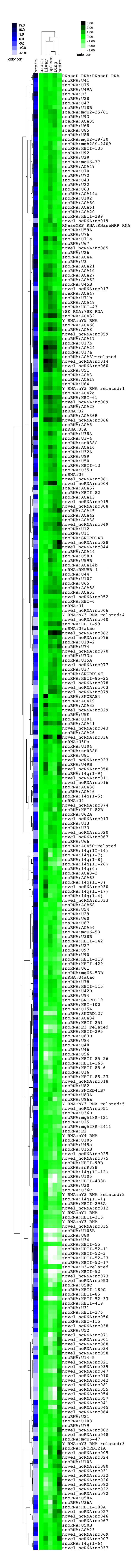

Supplement: Figure S5 — Clustered expression profiles of is-ncRNAs in different tissues. Expression patterns of 326 clustered ncRNAs (the figure includes both novel and known ncRNAs) in human fetal brain, liver, spleen, lung and heart tissues. (TIF) [file pone.0021652.s005.tif]

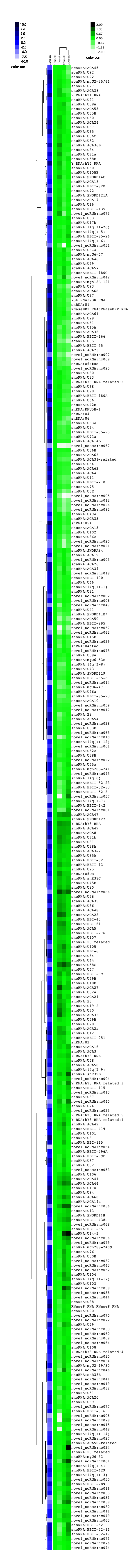

Supplement: Figure S6 — Clustered expression profiles of is-ncRNAs during human fetal brain development. Expression patterns of 326 clustered ncRNAs (the figure includes both novel and known ncRNAs) during human fetal brain development. (TIF) [file pone.0021652.s006.tif]

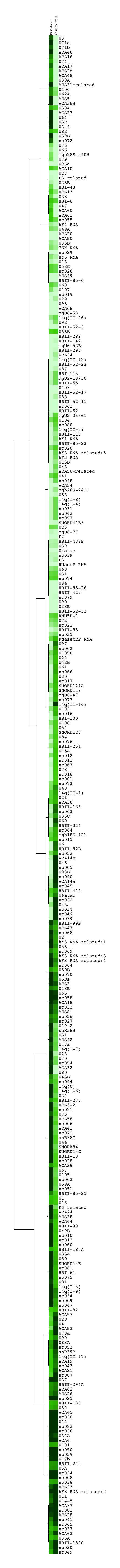

Supplement: Figure S7 — Clustered expression profiles of is-ncRNAs in tumor cell lines. Expression patterns of 326 clustered ncRNAs (the figure includes both novel and known ncRNAs) in glioma cell line U251 and neuroblastoma cell line SH-SY5Y, as compared with normal brain tissue. (TIF) [file pone.0021652.s007.tif]
