# Supplementary material for: Identification and Analysis of Intermediate Size Noncoding RNAs in the Human Fetal Brain
Source: PLoS One. 2011 Jul 18;6(7):e21652. doi: 10.1371/journal.pone.0021652 (PMC3138756; doi:10.1371/journal.pone.0021652)
Supplement: Table S1 — Distribution of sequenced clones. Distribution of sequenced library clones on different RNA species and categories. Sequenced clone numbers and percentage of novel and known ncRNAs are indicated in the table. (DOC) [file pone.0021652.s008.doc]

| Type of clones | Number of clones | Percentage of clones | Number of genes (percentage) |
| --- | --- | --- | --- |
| snRNA | 8516 | 41.46% | 9 (2.76%) |
| snoRNA | 6917 | 33.68% | 209 (64.11%) |
| rRNA fragment | 1634 | 7.96% | － |
| tRNA | 937 | 4.56% | － |
| hY3 | 797 | 3.88% | 6 (1.84%) |
| hY1 | 563 | 2.74% | 1 (0.31%) |
| 7SK RNA | 304 | 1.48% | 1 (0.31%) |
| mRNA fragments | 245 | 1.19% | － |
| hY4 | 199 | 0.97% | 1 (0.31%) |
| hY5 | 179 | 0.87% | 1 (0.31%) |
| RNase P | 63 | 0.31% | 1 (0.31%) |
| scaRNA | 45 | 0.22% | 14 (4.29%) |
| RNase MRP | 39 | 0.19% | 1 (0.31%) |
| **novel ncRNA** | **101** | **0.49%** | **82 (25.15%)** |
| total | 20539 | 100.00% | 326 (100%) |
