# Supplementary material for: Identification and Analysis of Intermediate Size Noncoding RNAs in the Human Fetal Brain
Source: PLoS One. 2011 Jul 18;6(7):e21652. doi: 10.1371/journal.pone.0021652 (PMC3138756; doi:10.1371/journal.pone.0021652)
Supplement: Table S2 — Genomic location of the novel ncRNA genes. The length and chromosome location of all the novel ncRNAs are presented in the table. (DOC) [file pone.0021652.s009.doc]

| ID | Length | Chrom | ChromStart | ChromEnd | Strand |
| --- | --- | --- | --- | --- | --- |
| nc001 | 125 | chr1 | 152745105 | 152745229 | + |
| nc002 | 54 | chr17 | 69137846 | 69137899 | ― |
| nc003 | 60 | chr3 | 52697948 | 52698007 | + |
| nc004 | 54 | chr13 | 43626696 | 43626749 | ― |
| nc005 | 128 | chr2 | 99624616 | 99624743 | ― |
| nc006 | 73 | chr5 | 88227534 | 88227606 | + |
| nc007 | 86 | chr4 | 142222518 | 142222603 | + |
| nc008 | 80 | chr7 | 28412851 | 28412930 | + |
| nc009 | 83 | chr1 | 245390922 | 245391004 | ― |
| nc010 | 94 | chr2 | 50887890 | 50887983 | ― |
| nc011 | 95 | chr7 | 8442902 | 8442996 | ― |
| nc012 | 85 | chr3 | 50257265 | 50257349 | + |
| nc013 | 66 | chrX | 114267136 | 114267201 | ― |
| nc014 | 72 | chr14 | 92973527 | 92973598 | + |
| nc015 | 94 | chr11 | 70125614 | 70125707 | ― |
| nc016 | 105 | chr16 | 6078344 | 6078448 | + |
| nc017 | 134 | chr17 | 57978231 | 57978364 | + |
| nc018 | 81 | chr12 | 49136755 | 49136835 | + |
| nc019 | 79 | chr7 | 150923121 | 150923199 | ― |
| nc020 | 65 | chr8 | 33330432 | 33330496 | + |
| nc021 | 71 | chr15 | 36793988 | 36794058 | ― |
| nc022 | 73 | chr1 | 50348085 | 50348157 | + |
| nc023 | 87 | chr7 | 44383468 | 44383554 | ― |
| nc024 | 81 | chr3 | 114733721 | 114733801 | ― |
| nc025 | 98 | chr11 | 56846856 | 56846953 | + |
| nc026 | 92 | chr3 | 136375094 | 136375185 | + |
| nc027 | 101 | chr1 | 148347550 | 148347650 | ― |
| nc028 | 59 | chr1 | 17104287 | 17104345 | + |
| nc029 | 149 | chr14 | 19861179 | 19861327 | ― |
| nc030 | 78 | chr15 | 60892201 | 60892278 | + |
| nc031 | 100 | chr1 | 14945409 | 14945508 | + |
| nc032 | 91 | chr8 | 10910850 | 10910940 | + |
| nc033 | 73 | chr1 | 236889127 | 236889199 | ― |
| nc034 | 73 | chr3 | 118767625 | 118767697 | ― |
| nc035 | 84 | chr1 | 159767606 | 159767689 | + |
| nc036 | 80 | chr3 | 69211322 | 69211401 | + |
| nc037 | 80 | chr16 | 84193817 | 84193896 | + |
| nc038 | 83 | chr18 | 68418875 | 68418957 | + |
| nc039 | 73 | chr1 | 83319897 | 83319969 | ― |
| nc040 | 61 | chr2 | 10242579 | 10242639 | + |
| nc041 | 68 | chr6 | 12858128 | 12858195 | + |
| nc042 | 67 | chr7 | 78919653 | 78919719 | + |
| nc043 | 52 | chr2 | 121549385 | 121549436 | ― |
| nc044 | 82 | chr2 | 233501022 | 233501103 | ― |
| nc045 | 73 | chr10 | 93506287 | 93506359 | + |
| nc046 | 99 | chr14 | 104952884 | 104952982 | + |
| nc047 | 74 | chr4 | 9746000 | 9746073 | ― |
| nc048 | 116 | chr5 | 179272369 | 179272484 | ― |
| nc049 | 81 | chr2 | 32881794 | 32881874 | + |
| nc050 | 116 | chr5 | 138639768 | 138639883 | + |
| nc051 | 85 | chr12 | 74762495 | 74762579 | ― |
| nc052 | 63 | chr1 | 32366477 | 32366539 | + |
| nc053 | 114 | chr14 | 35301761 | 35301874 | ― |
| nc054 | 92 | chr2 | 191500151 | 191500242 | + |
| nc055 | 77 | chr7 | 99786212 | 99786288 | + |
| nc056 | 88 | chrM | 13293 | 13380 | ― |
| nc057 | 69 | chr2 | 135288458 | 135288526 | ― |
| nc058 | 127 | chr11 | 72341502 | 72341628 | ― |
| nc059 | 72 | chr8 | 120215664 | 120215735 | + |
| nc060 | 67 | chr11 | 47508646 | 47508712 | ― |
| nc061 | 54 | chrX | 116938360 | 116938413 | ― |
| nc062 | 176 | chr6 | 165679944 | 165680119 | ― |
| nc063 | 71 | chr4 | 87452350 | 87452420 | ― |
| nc064 | 95 | chr9 | 110914259 | 110914353 | ― |
| nc065 | 62 | chr15 | 50284151 | 50284212 | ― |
| nc066 | 111 | chr13 | 93284952 | 93285062 | + |
| nc067 | 100 | chr3 | 54907945 | 54908044 | + |
| nc068 | 65 | chr5 | 115837908 | 115837972 | ― |
| nc069 | 92 | chr12 | 104002384 | 104002475 | ― |
| nc070 | 238 | chr12 | 115467056 | 115467293 | + |
| nc071 | 92 | chr13 | 107025722 | 107025813 | ― |
| nc072 | 97 | chr17 | 28630626 | 28630722 | ― |
| nc073 | 75 | chr2 | 207757574 | 207757648 | + |
| nc074 | 55 | chr20 | 34546587 | 34546641 | + |
| nc075 | 223 | chr3 | 197789831 | 197790053 | ― |
| nc076 | 71 | chr9 | 133596102 | 133596172 | + |
| nc077 | 264 | chr17 | 16328852 | 16329115 | ― |
| nc078 | 206 | chr4 | 151270558 | 151270763 | + |
| nc079 | 245 | chr1 | 238389640 | 238389884 | + |
| nc080 | 310 | chr5 | 145051566 | 145051875 | + |
| nc081 | 76 | chr3 | 121988603 | 121988678 | + |
| nc082 | 411 | chr14 | 69302948 | 69303358 | ― |
