# Supplementary material for: Identification and Analysis of Intermediate Size Noncoding RNAs in the Human Fetal Brain
Source: PLoS One. 2011 Jul 18;6(7):e21652. doi: 10.1371/journal.pone.0021652 (PMC3138756; doi:10.1371/journal.pone.0021652)
Supplement: Table S3 — Novel ncRNAs involved in axon guidance pathway. Four ncRNAs, their host genes and annotations are shown in the table. (DOC) [file pone.0021652.s010.doc]

| ID | NCBI number | host gene | annotation |
| --- | --- | --- | --- |
| nc012 | NM_002070 | GNAI2 | Guanine nucleotide-binding protein G(i), alpha-2 subunit (Adenylate cyclase-inhibiting G alpha protein) [Source:UniProtKB/Swiss-Prot;Acc:P04899] |
| nc026 | NM_004441 | EPHB1 | Ephrin type-B receptor 1 Precursor (EC 2.7.10.1)(Tyrosine-protein kinase receptor EPH-2)(NET)(HEK6)(ELK) [Source:UniProtKB/Swiss-Prot;Acc:P54762] |
| nc044 | NM_019850 | NGEF | Ephexin-1 (Eph-interacting exchange protein)(Neuronal guanine nucleotide exchange factor) [Source:UniProtKB/Swiss-Prot;Acc:Q8N5V2] |
| nc068 | NM_001080396 | FAM155A | Transmembrane protein FAM155A [Source:UniProtKB/Swiss-Prot;Acc:B1AL88] |
