# Supplementary material for: Identification and Analysis of Intermediate Size Noncoding RNAs in the Human Fetal Brain
Source: PLoS One. 2011 Jul 18;6(7):e21652. doi: 10.1371/journal.pone.0021652 (PMC3138756; doi:10.1371/journal.pone.0021652)
Supplement: Table S4 — Predicted snoRNAs or scaRNAs of novel ncRNAs. Fourteen ncRNAs with clear snoRNA or scaRNA characteristics were identified. As indicated, four ncRNAs were identified as C/D box snoRNAs, nine as H/ACA box snoRNAs, and one transcript (nc089) which showed both C/D box and H/ACA box characteristics is a likely scaRNA candidate. (DOC) [file pone.0021652.s011.doc]

| ID | original length | predicted length | snoScan/snoGPS | snoReport | snoSeeker | Result |
| --- | --- | --- | --- | --- | --- | --- |
| nc003 | 60 | 84 | C/D box | C/D box | C/D box | C/D box |
| nc016 | 105 | 102 |  | C/D box |  | C/D box |
| nc027 | 101 | 129 | H/ACA |  |  | H/ACA |
| nc029 | 149 | 148 | H/ACA |  | H/ACA | H/ACA |
| nc030 | 78 | 106 |  |  | C/D box | C/D box |
| nc031 | 100 | 128 | H/ACA |  |  | H/ACA |
| nc053 | 114 | 131 | H/ACA |  | H/ACA | H/ACA |
| nc058 | 127 | 137 | H/ACA |  |  | H/ACA |
| nc070 | 238 | 194 | H/ACA | H/ACA |  | H/ACA |
| nc075 | 223 | 130 | H/ACA |  |  | H/ACA |
| nc078 | 206 | 132 | H/ACA |  |  | H/ACA |
| nc079 | 245 | 141 | H/ACA |  |  | H/ACA |
| nc080 | 310 | 189 |  | C/D box |  | C/D box |
| nc082 | 411 |  | H/ACA | C/D box; H/ACA | | scaRNA |
