# Supplementary material for: Identification and Analysis of Intermediate Size Noncoding RNAs in the Human Fetal Brain
Source: PLoS One. 2011 Jul 18;6(7):e21652. doi: 10.1371/journal.pone.0021652 (PMC3138756; doi:10.1371/journal.pone.0021652)
Supplement: Supplementary Materials S1 — Oligos used and all novel ncRNA sequences. (DOC) [file pone.0021652.s012.doc]

**Oligos used:**

Underline: RNA; p.: 5' phosphate; -x: 3'-DMT

>5AD6; 5' adaptor

GGAGUAGCAUGCGUGACGAAAA

>3AD6; 3' adaptor

p.UUUUGACCACGAGCTCACAGGG-x

>5CD6; 5' PCR primer

GGAGTAGCATGCGTGACGAAA

>3RT6; 3' reverse transcption & PCR primer

CCCTGTGAGCTCGTGGTCAA

T7P-3AD2

5'-GGCCAGTGAATTCTAATACGACTCACTATAGGGAGGCGGCCCTGTGAGCT

CG TGGTCAA-3'

**All novel ncRNA sequences**

>nc001

TTTCCACAGGCCAGCCACTGTGCAGAGTGGTGTGTGGTTTTAAAGTTGCCCATGCTGTCTGTATTAGTTTTCGATTTGCTTTCATAACAAAATACTACAACCTTAGTGGGTTAAACAACGCAAAT

>nc002

TGAAGAGAAGGTGCTTCTCCTAGGGCCCGAGTCTAGGTGTCATGGTGGTGGCTG

>nc003

GAAATGATGAGGGTCAACATTCTTCATACCAAAGTGAAGACATGAGATCCAACTCTGAGC

>nc004

TGAAACAGCCTATTGTGTCAGAGAGGTGCAATAGTATGGCTGGGTCAAATTGGA

>nc005

AGAACAGTCGCAGGTCCGGTAGTGTACTGAGCATCTCACATACCTTACTTCATTTAGTCCTCGCGACGACTCTCTTGGTGAGGAAGGAAGAATGCAATGTGAGGGCCTTAAAGAGATATTTTCCATGA

>nc006

CATGCTTCCACAGATACCCTGCTGTGTTGGTCAGGTGGTGTTTTAGGGAGCACTTTCAGTATGTCTCAGGTCC

>nc007

CGTAAACTGTTCCACTTATGCCCTAGGGCTCTCAGAACTTTATGGTCACTCTGCATTCAATACTGTTTCACTGTCAAAGTATAATT

>nc008

AGCAACTGCAGCCTCCATGAAACTCTGGCTCTGAGAAATCCTGGGAGAGACTGAGTGAGCATGAGGCAGAAATGTGAGTC

>nc009

GCTGAACTGCTAGAAAGTCTGTTTCAGGCATCTATGAAATTTTACATAAATATCATTTGTAAGAGGTGACATCTAGATGACAT

>nc010

ATTAGTTAGTTGATCCCTTGAGTGGTCTACCTTAGCCATACCAAAGCTGGAATCTGTCATTAACTTGTCCCTGGTCATAATTATGAAGGTTATA

>nc011

AGGCTCTTGGGATTGAGGAGCGCCAGTATCACCTCCAATCAGAGGTGTAGCCCATCAGAGAGTTCGGGCTGCTTTAGCGCACCTCACCCCTAGAC

>nc012

AGAAGCGTCCAAGCCTCTCTGGGCCATGAAGCCATGCACAGAGCAGAGACCCGTGCATCCTCATGGCCACCCAAGACTGTGGGTC

>nc013

GAAATTTCCTTTTTCCACGAGAAACATCCCTGCAGTGGGTTGTTCCTATCGACAGGAGGTTACACG

>nc014

GTGTCATCCTTGCGCAAGGGTCATGCGAGTCTTTTGCATATCATTCTAATATTTTCAGTGAGCACTTACCCT

>nc015

TATCCAAACTGTATCAGCTGCGTTCCAGCCACCTGGGTGGCAGGTCTCCTGGCTCCATGCTGCAGCTGCCCCTGTTCTGGTTCAGACCTGGCAG

>nc016

CAGAGAAGTATGATTAGTCATGTTCTCTGATGTGATTTAGTAATTGTCATTTGTCTATTATTAGGTGAAATTTTCTAGCTGAATTTTTCCAAAGATGAATAAACA

>nc017

TTCATACTATAAACTATTGAAAATCAAGTGCTAAACTGATACTTTACTTTTAAAGTTGTTTATTGAATTATCTTTTAACTCTTATGATTAGGTATTGTTTTTTCGTCTTGGATTTTTCCTTTTGTGCTGTTGGG

>nc018

CTTCATTCTTTCTCTCTTTTGCCTGGATCGAGATTGTTAAGTCCCTCTCAGTGAAGGGTAAGATTATGAGATCTGAGGGCT

>nc019

TAAGAAATGTGCTATTACTGTCTTCTTCCTAAAGACAGGGAAGCTCAAATTATAGAGTTTAAGAACTTAAGTCAGAAAG

>nc020

TTTTGTCTGGAGATTCTGATCAAGTTGACACAGACTAAAAGTCTTTTCCAAGGTCATCCTAGAGG

>nc021

GGGGTCTGCAGGGGAGGAGAGCAGAGCTAGGAACGAGCTTCCTTTGCTGTTGGCTTCATCTCCAACCACAA

>nc022

AGTTGTTGCTTCCTTCTCTGGGCAGCAGCTCTGTAAGAAGGAATGTCAGCTTTTACATAACGTTCCTTTCCGC

>nc023

TGCACTCAACTGTGAGGAAGACACCAGCTGGGGGTCAGCTAGGCCCATCTCTTCTCTGAGAACTGACCCCCTCACAGGGATTGCCCT

>nc024

AACAATTTCCAGCACTGCCTGAAGTCTGGTCCCGGAAGTTGTCAAAAAACGACTCAGACTAACTAGATTTATGCAGAAGGC

>nc025

GTCAGGGTGGAGCAGCCACAGCCTATGTATGAAAAGGGGTTATGTGGGCTTCACTCACCAGCCTAGGGTTGGCTTTAAGGAGTTCTGAGGGGGACAGA

>nc026

CCCTGCTCTCATGGAAAAAGACAACCACCCAGTGTCCTGGTCTTAGCCCTCAGAGGTCGGTGGGGCAGACTATTTCTAGTGCTCGTCAGCCT

>nc027

TCCGTGGTCTGTTAGGAACTGGGCGGGGGCACAGCAGGAGGTGAGCAGTGAGTATTAGGGCCTGAGTTCTGCCTCCTCTTAGATCCGTGGAGGCGTTAGAT

>nc028

TGTAGAGCACATGTTATGGTTATCAAAGTGGTTTACTGTAAGGCTGAGGGCTATGCATT

>nc029

TTTGATGGCTGTTCCTCTCACTGCTTGAAGCCTTAGGCAGTGGGATTTTGATCCATCATATATCAAAAATGGCTTATCTTCACTCAGGGCACCATGAGGATGGGCTGGCTGTCCGTTAGTGCCTTCTGATTTTTGCGGAGTCAAACAAT

>nc030

CAGCCCTGATGGTAAACGGCAGCTTTTTCTGTAACAGGAAGAAGGGCACTTACTGACGGGAGACCCACCATCACAGGT

>nc031

AACATGTTACTGAGTGGCCTCTTCTCCACCACTCAATAACAAGAGCAAGTATTTTGGAAGGATTAAAAGTGCGGAGGACTCAGTCTCTGCTGCAGGTTTC

>nc032

CCCGACTCCTGCGGACTCGGCTGCAGGCGGGCTCCATGAGGGCGGGCAGCAGGGCTCAGACGATCCGGCTCGGTTTGGTCAGCAGACCCCC

>nc033

AAGAAGGAAGTCACTGTTTGATGTAAACTAAAAATGAAGTCTAAGCTCCCTACTAGCTGAAAATGGACCCCCT

>nc034

CTCTCACCCTACTGCCCTTTTCTTTCACCTCCTGCTCAGTCTGACTGCCCTCCATTACTAAGCTGCATTCTTC

>nc035

TTGTCTTTCCTTCTACGAAAAACCTTTCTCAGTCAGAACCTATAACGCGCTGTGTATCCGTTTTACGCCTCAACGAAGTATCCG

>nc036

TGGCGCTGGCCAGGTTAGAAAACACTTCGATTAAATCTGTCTCACCAGCATAGAATTAAGCATCTGATAAGAAATAGGGT

>nc037

AGCCGGTTGATCTTTATGAAGGAAGACAGTTCCTCCCGGGCTTTTGAGTGAATCACCCAAATTTCCGGTCCACTGGTGTC

>nc038

AGACTGCAACACTGCAGTAAAGAAAGAGTTAAATTGACATGAGGGCAGTCACATAGGAGAAGGGGTTATTACTCAAATCAGTC

>nc039

CACTCTTTTCTACTTCTATTCCATTCTCTTCACAGCAACTAGAGTGGTTATAATGATTGCTACCTTGCTTGAG

>nc040

ATCTGACAAATGAACAAGTCATTCTATTTACTGGTTTCTTTGATCTATTTATAAAAATGAA

>nc041

CTCGCGCTCTTTGTGTCTCCGGTGCGCAGAGGGCGTGTGTGCGAGCTACGGGGTGGGGGAGGGCGGCG

>nc042

ACACGGAGTGAAGAAGGGGAAAAGTTACAGAAGACCCGTCAAAGCGCCCAAAGCAATTTGTCCCTTC

>nc043

TATTTTTAAAGCGTATGATTAATACACTTGCCTATTGCTGCGTGGACTGTGC

>nc044

GCTCGGCAGCGGTGGCGGCGGCGGCTGCAGCTCCGGTCGAGCCGCGCGCGCTGCGACCCCGCTCCGCAGCCCGCTAGTCGCC

>nc045

CATCTGGTGAGAGCTTTCTTGCTGGTGGGAATCCTGTACAGAGCCCAGTGGTGGTACAGGAGATCACATGGTA

>nc046

ATGGAAATACAGAAATACGTGTCGAGTTGCTACTTATAGACTTTTAAATATTTAAAGAAAAAAATTGCTAAAAGCAGATGAATGCTTCTTCACCTCAAG

>nc047

TCTGCCTAGAAGGGGGTCTTTCTCAGCATCATGAGCTGGCTTCATATCCTTCATGGGAAGGGGCCATCCAAGCC

>nc048

CCCAACACAGAGTGAATTCAAATTTGCTTTTGGAACAGCCTTTATCTCCAATTAAGTGCAAGTATATTTACACTATGTGATATTAAAGGACTTCTCCAATGACTGGGAGGTACTGA

>nc049

TAACCTCAGTGAGTGGAGGGCAAAGTAGAAGGGCACTCTTAGGCGCCGGCCAGGCTGCCCAAGCCCTTGCTGTCTGCTCTC

>nc050

TCCAGCTGTAGGCAGCTGCCTAGGTTGTCTTGTACCTAGGCAAGTGTTACACTGCTGGGAGAACAGCAGCCAATAGCTGGTTGGCATTCTGGCCCTGGTTCATGCCAACTCTTGTG

>nc051

TAGTAAATAGAAATTTAAAAGTTTTCACTATACCAATTTGAATATCTTTCTAAATTTTATCTGCTACAAAGTGCCTATTTTTATA

>nc052

CACTCAAGGGCCAAAGAACTAGTAAATCCTAAGAGAGAATTAGGATTAGCAAACTAGAAGAAC

>nc053

ATATGCCCTTCTGATCCCAAGTATGGCAGAAGGATATAAAGGTCACCAAGAATAATTTGGAGGTTGCCAAGGACTTATTTAAAAGTGTTTTCAAGTCCGACCGAGAACTCTGAC

>nc054

TATTAAAGTAATCTCTTTGGAAGAAATATTTCAGATCTGTATGAAATACCAAATAAAATAGATTATCTTAAAAGTGAACAGTAATATTAAAA

>nc055

TGACTTGGGACAGGGCTGTCTCTTCAGTCCTTACCACACCGTGGACGAGATCAACCTTAAGACAGCACCCTGGGCTC

>nc056

TGGATGATGGACCCGGAGCACATAAATAGTATGGCTTTGAAGAAGGCGTGGGTACAGATGTGCAGGAATGCTAGGTGTGGTTGGTTGA

>nc057

TGTAATTTCTAAGAAACTCCAACTTGTATTTCTTAAACACCATTTAGGGGACACATAGACCAAAATAAA

>nc058

AACTGCTGACCTTGAGGAAAAGCAGATAATTCTATCAAGAAAGCCTCCCTCCAGGAGTAGGCTTTTATTGCTTTATTATATATCTTAAACTGTAAGATCCTGTTTCTTCACCTGTGGAAATAAGGTG

>nc059

GACTAAGGCAAGAAGTGCAAAAGTTTATGTGGTTAGGCTATATAGTCTGATAAACTGTTATCAAAATGAATT

>nc060

AACAGCTATATTCTATAAGCTGCTACCAAAGAGAGAAACATCATCCTCTCTAGACTGCCGTGGAGTC

>nc061

TTGTAACCTCATTTTACCTTGATTAGTGACTCAACTCATGTTCTTGTTGTAGGG

>nc062

ATTTTGCCTTCCTTTGGTAAAGTTCTGTGATTCCATGATCTTCTGATGTGGGAAGAAAATCTTGAAATAGGATGGCACTTAGCAGGCAAACATGCACGCTGCGGAGAGGTGCCCGCCTCTTTGAGGTTGGAATATCTGCACCCCTGTGATAGAGCCTGTGCTCTTTATCTGCTCCA

>nc063

AGTTCTGTCCAAAAATAGAAGAAATTGTTTCATGTGGTCACAAGCTGCTTGTCACTAGAAGTATTTAAACT

>nc064

TCTTTTTAATACTGCTACAGTATAAATCTTTTTTCTCCCCCTCCCCCACCATGCCTTTTAGAAAATGTTTTCAACAGATTTTCTTAGCTCTGAAT

>nc065

CCCTAGGAGCCCCGGGATGCTGGCGCCCAGGGCCTACTCCCCTCACGCCACACCCTGCCAAC

>nc066

TACCTGTGTAACTACAATTGAGTCCCTGAGCTACCCTCAGTTGTTTGTAGTACTAACTACTGTTCATGGACCTGCTAGCAAGTCAGTTTCCATGTTCCATATTGTGCTAAG

>nc067

CACCAACTGCAAGTCTCTGTGGTGGAGCTCTCTCTGATTTTCTCTGCTATGTGTTGAATACCTGTACAAACTAGAAAAGAAACTGGTTTTTATTGCTGCT

>nc068

TTTGATCAGTGGACCTTAATCATCTTGGTGCATTATAGCTTTATTTGCAAGGAAACAGGTTTTAA

>nc069

GAGAGCGGCGAGCCGCGAGCCAGGCAGTCCGGGGCATCCAGACTGCAGGCCGCGCCCAGGCCGCGCCCAGGCTGCGCCGCCCGCCTGCCTCC

>nc070

ACACAGCAACTGTGGAACAGTTTTGTGATTTATTCAGAAAACTGGCTCCAAGATCGCTGTGGACAAGACCCAACTTGGCTGGGAGATTGTAATCCCTCAGTAGAGCCACGTGGACAGTGAAGCTGGCCGAAGTTTACTCTGAGAAAGGATCAGAGACTGAAATTGCTCTGATTTTCCTAAACCCAGCTTCTGTAGTCAGGAAGAGGTAACTGTCATATTTAAAGGCAAGTACTTTCAG

>nc071

GGACTCTCTGCTAAAATTGTAGAAAGGGGCTCATGGGATGGTGCCCTGGAGAGACAAGAGCCTTTCTGGAATGGGCTCTGGTTCTAGTTCCT

>nc072

ATGTTGACCCCTGGCTACCTCACTCCCATTGCCTCTCTCTCGTGCTCCACGTTCCAAGAATTTTAACCAGCCAACAAGCCAGGAAAGCACCTAATAC

>nc073

TTAACCCCTCCTAGGAAATATCCCTTCCCATAGCACTTCAGAGTCTTTGCGGCAACTCTGCTGCAATGTCCCCAC

>nc074

AGGAACAAGGAGGTCTGGGGCAGCTGCTGCGGCAGTGATAGACTCGTGTGCCATG

>nc075

ATAATGTTTTAAGAAAGTTTATGAATTTGCATTGGGTCACAATCAAAGCCATCTTGGGCTGCATGCAGGCTGTGGGTTGGACAAGCTTGATATAGAGCATGAACTGGCAAACTACAGCCCCTGGACCAAATCTGTCCTGCAGCCCATAAATAAGGTCTATGGAATACAGCCATGTTCGTTCATTTACGTATTATCGATGACTGCTTTTGTACTATAAATAGAG

>nc076

TTCTGAAGGAATCCTCAGGATGATGGGGTGTCCTGTTAGTGCAGCAGCTGCCTCTGTGGCAGAGGGGTCCC

>nc077

AGGGGCGGGGACCCCCTCCTATGGTGCCTGCTCTCCTCCCTACCCCCACCCGGCCCTGAGCAGTGCCCACGTGGCTCTCCAGGAGCATTTCCCAGCTTTTTTTTTTTTTTTTTAACTTTTATTAGCTCAGACTCCTGGCTGCTGCAGCCATATGGAGCTCTGCCGACGGCTGCTGCTCAAAGAGATGTGGCATTTTGTTGTTTCTAACTTTATCTTAACCAGGAGAGACACAGGAGCACAGCTGAGAGTGGGGAG

>nc078

TTTGGTGACATTATATTGTATAATGGTTCCTGGTTAATGGCTTATTTGAGCTATATCTAGAGCAATTTAGCACTGATACTGTATAGGAAAATGAGAGAACGGGATTAAATTCGTGGATGTTTTGCACGGAAGTTTTTAATAGGATAATTTATTTGAGCAAACATCCAGTCTATTTAATGAATCTGGAATGCTGTGGAGCTCAGAAG

>nc079

CCATTTATTACTTTTAATTCTAATTACAAATAATGCATGCTCACTGAGAGGGTTTGAAAGTTTATTTTAAAAAGCCCAAGTCATGGTGGTATGAATGTTTTTGCTCTCTCTTGAGTGCTTAGATTTTCAATCCCTCTTGGAGCATATGAATTATTTGCATACATGGAGAGATATATATATATATCTGTGTATATATATATATATATATATATATATTTGTGTATATATATATATCTATATCTGTG

>nc080

TGGAGCCCAAGATTGACCAGAAGAAGGGCTACTTCTCTAGCACCTCAGGATCCTGTCTCCAGCAGCACAGAACACCCTACTGAATACCCTGAATACTCCATGCTGTTTCATGCCTCTGTGCCTTTGACCACCTTCTTCTTTTGTCTGGAATACCACCCTGGCCACCTGATGAATGCATCCTTCAAGACCTATCACAAGCGAGTCCTCCTCTATGATGCTTGCTTGAATCCCCTCCCTCCCTGATTGCAGCTGATG

>nc081

TGAATGAGTGAGGGAGAAATAATTCATTCATTTCATAACTTGTTGGTATTATCTGAGACAAGTGGACACTGAATGG

>nc082

GACGTCGTTCGCCCCTGCTAAGTCCTTGGCGTTCGGTTCATCATCATCTCTCCCAGAAGAGTCTCCTGAGAAAAGTGGCAACCCTTCTTGTATCTAAAGTTTTTCTTAGACACCCAGAAGATTACTAATTAGGAGAAACTTAGCTTTGCAGAAATACCTGGCCTTTCCTTAGCTCTTGCTGCTTGCAATTTAAATTTGCCCTCAAAGCAGGGAGCTCCTAGATGTTCGAGTTCTTTTTTCAACCATGCCACAGCT
